# Unique case study: Impact of single‐session neuromuscular biofeedback on motor unit properties following 12 days of Achilles tendon surgical repair

**DOI:** 10.14814/phy2.15868

**Published:** 2024-01-09

**Authors:** Carlos De la Fuente, Rony Silvestre, Julio Botello, Alejandro Neira, Macarena Soldan, Felipe P. Carpes

**Affiliations:** ^1^ Exercise and Rehabilitation Sciences Institute, Postgraduate, Faculty of Rehabilitation Sciences, Universidad Andres Bello Universidad Andres Bello Santiago de Chile Chile; ^2^ Unidad de Biomecánica, Centro de Innovación, Clínica MEDS Santiago Chile; ^3^ Foot and Ankle Surgery Department Clinica MEDS Santiago Chile; ^4^ Carrera de Kinesiología, Departamento de Ciencias de la Salud, Facultad de Medicina Pontificia Universidad Catolica de Chile Santiago Chile; ^5^ Escuela de Kinesiología, Facultad de Medicina y Ciencias de la Salud Universidad Mayor Santiago Chile; ^6^ Escuela de Kinesiologia Universidad de los Andes Santiago Chile; ^7^ Laboratory of Neuromechanics Federal University of Pampa Uruguaiana RS Brazil

**Keywords:** case‐report, decomposition, neuromechanical adaptation, surface electromyography, rehabilitation

## Abstract

We explored the first evidence of a single‐session neuromuscular biofeedback effect on motor unit properties, neuromuscular activation, and the Achilles tendon (AT) length 12 days after undergoing AT surgical repair. We hypothesized that immediate neuromuscular biofeedback enhances motor unit properties and activation without causing AT lengthening. After 12 days AT surgical repair, Medial Gastrocnemius (MG) motor unit decomposition was performed on a 58‐year‐old male before and after a neuromuscular biofeedback intervention (surface electromyography (sEMG) and ultrasonography), involving unressited plantar flexion. The analysis included motor unit population properties, sEMG amplitude, force paradigm, and AT length. There were increased MG motor unit recruitment, peak and average firing rate, coefficient of variation, and sEMG amplitude, and decreased recruitment and derecruitment threshold in the repaired AT limb. The non‐injured limb increased the motor unit recruitment, and decreased the coefficient of variation, peak and average firing rate, inter‐pulse interval, derecruitment threshold and sEMG amplitude. The AT length experienced −0.4 and 0.3 cm changes in the repaired AT and non‐injured limb, respectively. This single‐session neuromuscular biofeedback 12 days after AT surgery shows evidence of enhanced motor unit properties and activation without signs of AT lengthening when unresisted plantar flexion is performed in the repaired AT limb.

## 1 INTRODUCTION

Immediately after an Achilles tendon (AT) rupture surgical repair, the ankle is usually immobilized at a shortened length for 4–6 weeks (De la Fuente, Peña y Lillo, et al., 2016). Due to the high risk of AT lengthening, expert panels recommend immobilization in the early phase of tendon healing (Saxena et al., 2022). Unfortunately, immobilization reduces triceps sural strength by decreasing motor unit discharge rate and recruitment (Duchateau & Enoka, 2017). Only 72 h of immobilization reduces motor excitability and recruitment, resulting in ~22% reduction of the maximal muscle strength (Duchateau & Enoka, 2017). A longer immobilization, for 4–6 weeks, leads to persistent impaired muscle activation up to 1 year after rehabilitation (De la Fuente et al., 2023).

Despite the high risk of AT lengthening (Saxena et al., 2022), neuromuscular feedback strategies using real‐time ultrasonography (US) and surface electromyography (sEMG) are potentially beneficial in early rehabilitation if administrated under controlled mechanical stimuli over the AT during healing process. Therefore, neuromuscular biofeedback could be implemented considering low‐intensity contractions, since repetitive loading has been tested without generating clinical failure (De la Fuente, Carreño‐Zillmann, et al., 2016; De la Fuente, Carreño, et al., 2017; De la Fuente, Cruz‐Montecinos, et al., 2017) for a model similar to an early stage of tendon healing (Freedman et al., 2017). This approach can increase the firing rate of active populations of motor units and the number of recruited motor units (Stein et al., 1990). These last neurophysiological stimuli would be relevant for improving immobilization impairments like muscle inhibition and persistent weakness (De la Fuente et al., 2023). However, whether motor unit properties can be enhanced during a single session of neuromuscular biofeedback without AT lengthening during the early AT healing phase following a surgical repair remains unknown. Understanding the physiological responses should be the first step before planning clinical trials.

Here, we investigated whether a single‐session of neuromuscular biofeedback intervention can influence Medial Gastrocnemius (MG) motor unit properties, neuromuscular activation, and the AT length only 12 days after AT surgical repair. We hypothesized that a single‐session of neuromuscular biofeedback intervention improves the MG motor unit properties and neuromuscular activation without increasing the AT length 12 days after AT surgical repair.

## 2 MATERIALS AND METHODS

### 2.1 Case description and design

In this case report, we compared MG motor unit population properties (number of recruited motor units, peak and average firing rate, coefficient of variation, decomposition accuracy, peak and average motor unit action potentials (MUAP), inter‐pulse interval, and motor unit recruitment and derecruitment thresholds) (De Luca et al., 2006; Jeon et al., 2020; LeFever et al., 1982), sEMG amplitude, force paradigm, and AT length before and after single‐session of neuromuscular biofeedback (US and sEMG biofeedbacks). The intervention included submaximal muscle contractions (30% of maximal voluntary contraction of the non‐injured limb) in the repaired AT and non‐injured limb of a patient on day 12 after the AT repair surgery (Table 1). The participant was a male recreational soccer player (age 58 years old, body mass 94 kg, height 1.86 m, and body mass index 27.2 kgm^−2^), an office worker without comorbidities, and who suffered a non‐contact AT rupture during a recreational soccer match. The rupture (acute, unilateral, and mid‐substance) was repaired with the triple Dresden technique by JB (De la Fuente, Carreño, et al., 2017; De la Fuente, Cruz‐Montecinos, et al., 2017).

**TABLE 1:** Basal measurements of the study.

Measurements	Non‐injured	Repaired AT	Δ
Achilles tendon resting angle (°) (Carmont et al., 2015) MG resting pennation angle (°) MG resting thickness (mm) Maximal voluntary isometric contraction (N)	14.0 18.0 17.4 171	12.0 20.0 16.3 –	−2.0 (−14%) 2.0 (11%) 1.1 (6%) –

Clinical scores	Obtained points/maximal points
Achilles tendon rupture score (pts)	87/100
Verbal numerical rate pain scale (pts)	
Prior to the intervention	0/10
Maximal value during the intervention	3/10

Abbreviations: cm, centimeters; MG, medial gastrocnemius; mm, millimeters; N, Newton; pts, points; °, sexagesimal degrees.

### 2.2 Neuromuscular biofeedback intervention

The neuromuscular (US and sEMG) biofeedback was delivered with the patient in a prone posture, performing 20 dynamic plantar flexion contractions without external resistance. The patient developed 20 s in total for each ankle plantar flexion, 5 s were used to move from the rest position to full plantarflexion, 10 s were used to maintain the full plantarflexion, and 5 s were used to move from full plantarflexion to the rest position. The participant repeated five series with 30 s of rest between series. Due to protective reasons, we used low‐speed ankle movement because high‐speed joint movement required higher stiffness material properties, which contradicts the rehabilitation principles of the early stage of tendon healing (no overload). The protective range of motion was defined from rest plantarflexion to full plantarflexion range of motion, in coherence with past recommendations for the early stage following the Dresden surgical design, which also was tested at low‐speed traction load (De la Fuente, Carreño, et al., 2017). A “beep” sound metronome was set. The patient was instructed not to talk during the execution of the exercise. The verbal instructions at the beginning of the exercise were: “Please move your (big toe/little toes/ankle in plantar flexion) in sync with the metronome's rhythm. If you experience any pain while performing the exercise, you must stop immediately and inform me to halt the assessment.”

**FIGURE 1:**
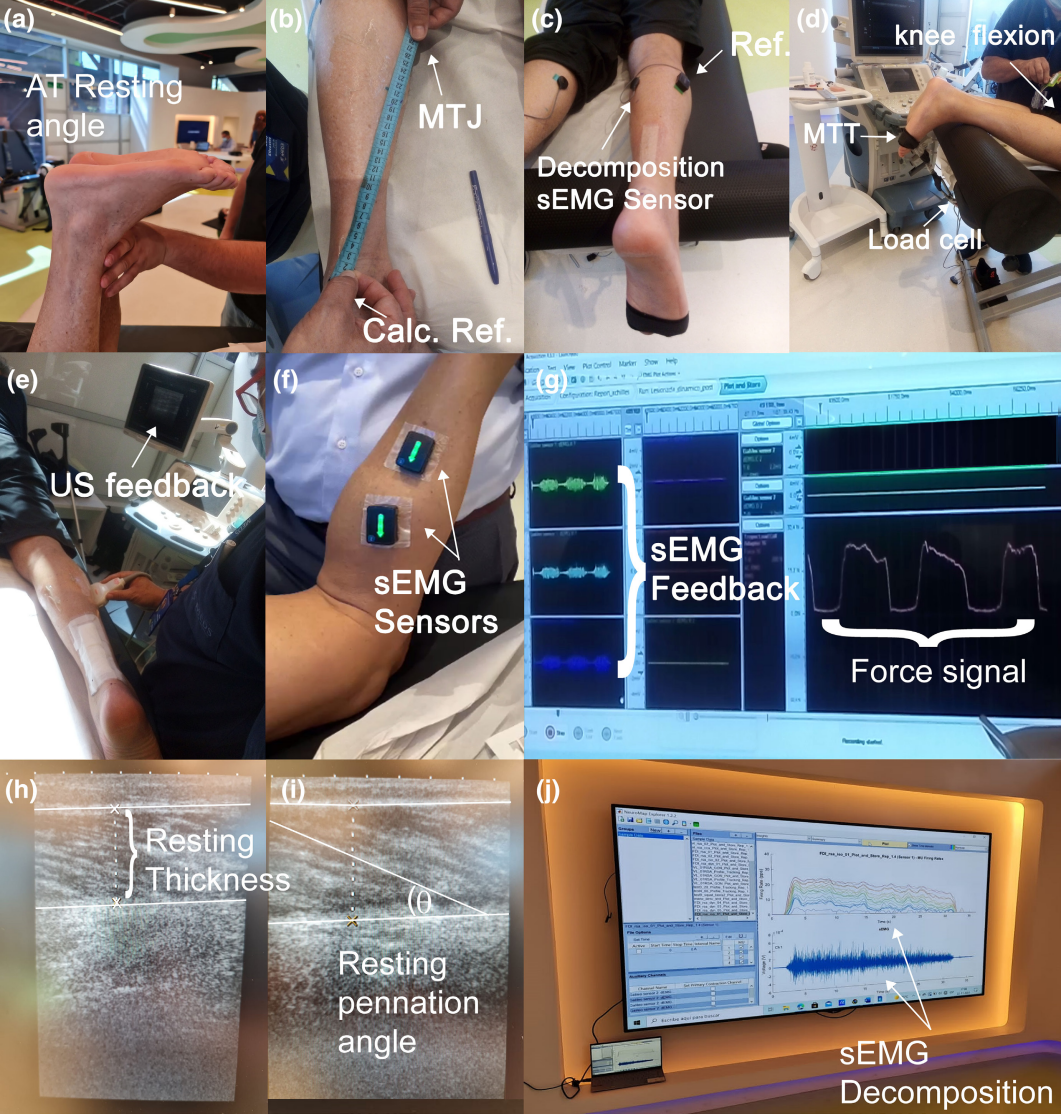
Biofeedback setup. (a) Achilles Tendon (AT) angle at rest. (b) AT length measured from calcaneus reference (Calc. Ref.) to the myotendinous junction (MTJ) of Medial Gastrocnemius. (c) EMG sensor on Medial Gastrocnemius to acquire firing rate during prolonged periods. (d) Measurement setup. (e) Ultrasonography feedback. (f) Surface EMG sensors for feedback. (g) Force and EMG signals projected on the screen. (h) Ultrasonographic measurement of muscle thickness. (i) Resting pennation angle measurement (Carmont et al., 2015). (j) EMG decomposition.

Both US and sEMG signals were projected on a screen in front of the participant, providing neuromuscular biofeedback for both the evaluator and the participant (Figure 1). Before testing, the evaluator guided the patient familiarization with feedback for 5 min. The US feedback was delivered in real‐time (Cho et al., 2014) (immediately visually projected) with the transducer positioned perpendicular over the third lateral portion and the third medial portion of the leg to identify the hallucis flexor longus (De‐la‐Cruz‐Torres et al., 2020) and flexor digitorum longus muscles (Mickle et al., 2013), respectively, for details see Suplementary Material. A good quality acquisition was considered when the displacement of the hallucis flexor longus and flexor digitorum longus muscles were distinguishable by both the evaluator and the patient during metatarsophalangeal flexion. The patient was requested to focus on the muscle displacement (aponeurosis movement represented by white pixel displacement) on the screen as an indicator of muscle activation while moving their big toe or little toes to elicit the targeted muscles (Figure 1).

The sEMG feedback was provided through the use of two sensors attached to the MG and soleus muscle bellies (Hermens et al., 2000) (Avanti, Trigno, Delsys Inc., Boston, USA), see Figure 1. A good quality acquisition was ensured by the signal‐to‐noise ratio of MG and soleus. The activation was clearly identified as at least double the width of the resting signal. The patient was instructed to focus on the increase in signal amplitude compared to the basal signal on the screen, which served as an indicator of muscle activation (Figure 1).

## 3 RESULTS

**FIGURE 2:**
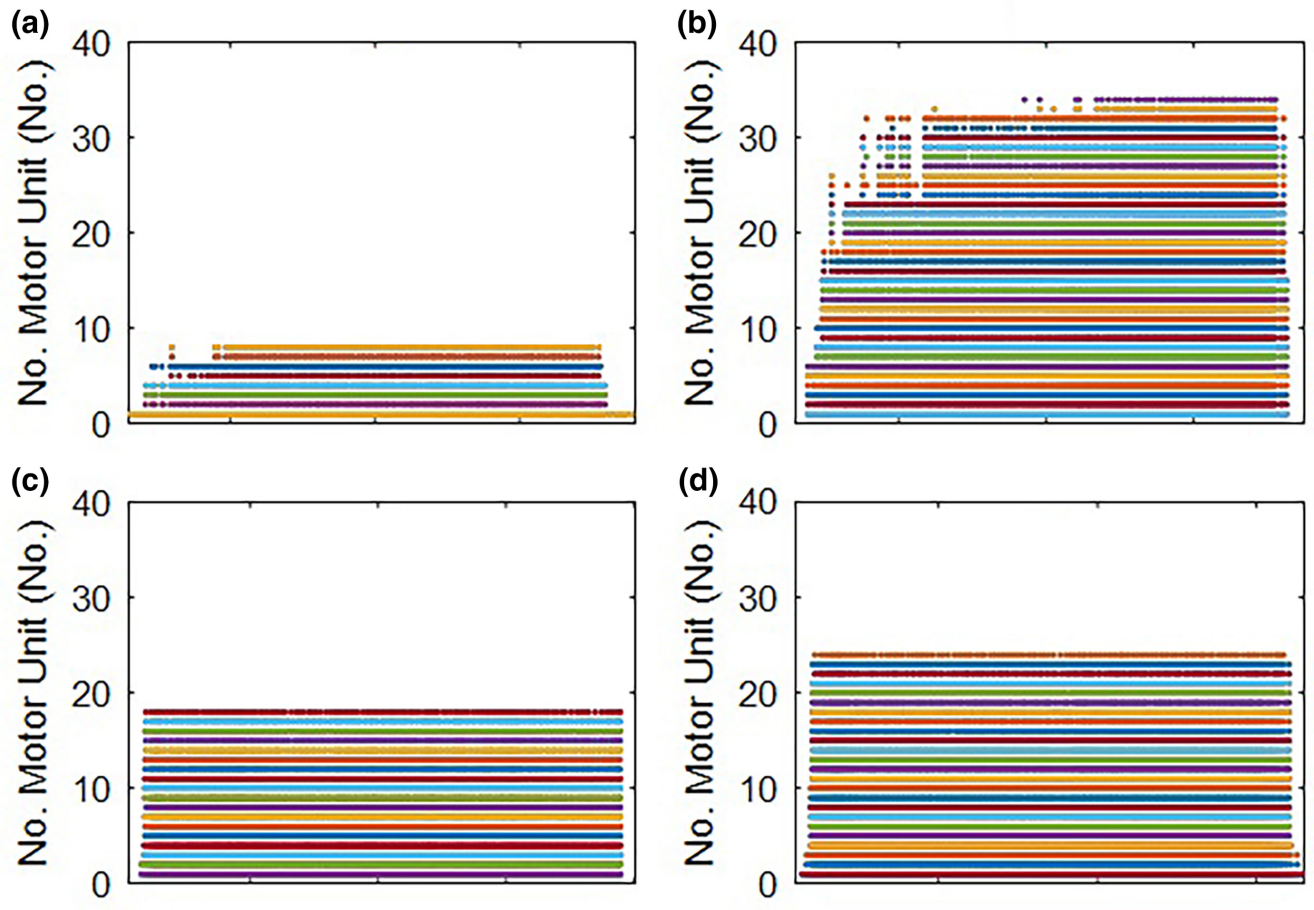
Raster plot of medial gastrocnemius motor unit recruitment before and after the neuromuscular biofeedback intervention during a single‐session. (a) Repaired AT limb before intervention. (b) Repaired AT limb after the intervention. (c) Non‐injured AT limb before intervention. (d) Non‐injured AT limb after the intervention.

The main changes observed following the neuromuscular biofeedback in the repaired AT limb were an increased number of motor units (Δ = 325%), peak firing rate (Δ = 74%), average firing rate (Δ = 40%), coefficient of variation (Δ = 40%), and sEMG amplitude (Δ = 52%, 43%, 26%, and 33% for each channel, respectively). In contrast, decreased derecruitment (Δ = −72%) and recruitment (Δ = −35%) thresholds were found in the repaired AT limb (Table 2; Figure 2). Decomposition accuracy, peak and average MUAP amplitude, and AT length showed changes lower than 10%.

**TABLE 2:** Motor unit and sEMG descriptive statistics, and cluster firing rate‐recruitment threshold.

	Non‐injured			Repaired AT		
Measurements	Pre‐BF	Post‐BF	Δ	Pre‐BF	Post‐BF	Δ
Recruited MU number (*n*)	19	24	5 (26%)	8	34	26 (325%)
Coefficient of variation (%)	4.2	1.9	−2.3 (−55%)	3.0	4.2	1.2 (40%)
Paradigm force (N)	31.0 ± 1.3	26.1 ± 0.5	−4.9 (−16%)	30.2 ± 0.9	24.0 ± 1.0	−6.2 (−21%)
Decomposition accuracy (%)	92.8 ± 20.9	92.6 ± 18.8	−0.2 (<−1%)	90.5 ± 28.8	86.5 ± 15.9	−4.0 (−5%)
Peak MUAP amplitude (10^−5^ V)	20.62 ± 22.66	10.70 ± 5.18	−9.92 (−48%)	3.59 ± 1.50	3.67 ± 2.53	0.08 (2%)
Average MUAP amplitude (10^−5^ V)	13.33 ± 12.07	7.70 ± 3.43	−5.60 (−42%)	2.95 ± 1.18	3.03 ± 2.32	0.08 (3%)
Peak firing rate (pps)	14.62 ± 5.96	14.57 ± 5.25	−0.05 (< −1%)	8.05 ± 3.05	13.99 ± 4.10	5.94 (74%)
Average firing rate (pps)	3.48 ± 1.99	4.57 ± 2.01	−0.05 (< −1%)	2.30 ± 1.32	3.22 ± 1.39	0.92 (40%)
Inter‐pulse interval (ms)	298.6 ± 147.2	153.4 ± 42.7	−145.2 (−49%)	201.1 ± 76.2	194.5 ± 68.0	−6.6 (−3%)
Recruitment threshold (N)	1.5 ± 0.4	5.0 ± 1.2	3.5 (233%)	22.0 ± 11.0	14.4 ± 10.3	−7.6 (−35%)
Derecruitment threshold (N)	26.8 ± 8.3	1.2 ± 0.3	−25.6 (−96%)	5.3 ± 2.8	3.1 ± 5.8	−2.2 (−72%)
sEMG amplitude						
rms electrode 1 (10^−5^ V)	1.0954	0.3803	−0.7151 (−65%)	0.9367	1.4247	0.4880 (52%)
rms electrode 2 (10^−5^ V)	1.0206	0.2295	−0.7911 (−78%)	0.8923	1.2774	0.3851 (43%)
rms electrode 3 (10^−5^ V)	1.2884	0.9226	−0.3658 (−28%)	1.1973	1.5074	0.3101 (26%)
rms electrode 4 (10^−5^ V)	1.1339	0.6543	−0.4796 (−42%)	1.0254	1.3636	0.3382 (33%)
Achilles tendon length (cm)	21.0	21.3	0.3 (1.4%)	22.5	22.1	−0.4 (−2%)

	Non‐injured		Repaired AT		Peak firing rate (pps)	Recruitment threshold (N)	Number of motor units (No.)
Clusters	Pre‐BF	Post‐BF	Pre‐BF	Post‐BF			
Cluster 0	0	1	1	2	7.4 ± 3.4	4.4 ± 2.0	4
Cluster 1	0	0	3	0	9.0 ± 0.3	22.0 ± 0.9	3
Cluster 2	2	22	1	18	15.9 ± 3.5	10.1 ± 6.8	43
Cluster 3	0	0	3	0	6.4 ± 1.2	30.6 ± 1.1	3
Cluster 4	0	0	0	14	12.9 ± 1.7	12.8 ± 1.7	14
Cluster 5	17	1	0	0	14.6 ± 5.3	1.5 ± 0.2	18

*Note*: Data are described with frequency, percentage, and mean ± standard deviation.

Abbreviations: ms, millisecond; MU, motor units; MUAP, motor unit action potential; N, Newton; No, number; Pps, pulses per second; RMS, root mean square; sEMG, surface electromyography; V, volt.

Regarding the non‐injured limb, the neuromuscular biofeedback increased the number of motor units (Δ = 26%) and recruitment threshold (Δ = 233%). In contrast, there was a decreased derecruitment threshold (Δ = −96%), sEMG amplitude (Δ = −65%, −78%, −28%, and − 42% for each channel, respectively), coefficient of variation (Δ = −55%), inter‐pulse interval (Δ = −49%), peak (Δ = 74%), and average (Δ = 40%) MUAP amplitude in the non‐injured limb (Table 2; Figure 2). Decomposition accuracy, peak and average firing rate, and AT length showed changes lower than 10%.

In total, we identified five different clusters of motor units based on peak firing rate and recruitment threshold space for both limbs (Table 2), and the AT length changed to 0.3 cm and − 0.4 cm for the non‐injured and repaired AT limbs, respectively.

## 4 DISCUSSION

In this study, we provide insights into the enhancement of muscle activation after a single‐session of neuromuscular biofeedback only 12 days after AT surgical repair, during the early stage of AT healing. Our intervention included a unique case study conducted in a typically restricted phase for research due to the high risk of adverse events (re‐rupture and tendon lengthening). To the best of our knowledge, this is the only study in which impaired motor unit properties acutely enhance after low‐intensity contractions and neuromuscular biofeedback without tendon lengthening. The repaired AT limb exhibited the recruitment of newly low‐threshold motor units, an increase in firing rate and sEMG amplitude, a decrease in derecruitment and recruitment motor unit threshold, and an increase in force fluctuations (higher variation coefficient). In contrast, the non‐injured limb showed reduced demands on motor unit properties to perform the task after the neuromuscular biofeedback. Therefore, we hypothesized that these strategies might have been influenced by improvements in: (i) spindle afference excitability, (ii) the net descending excitability input, (iii) motor unit synchronization, and/or (iv) short‐term potentiation on α‐motor units.

The improvements in motor unit properties indicate that the gamma loop of the plantar flexors is involved in force production. This assumption relies on the fact that low‐intensity muscle contractions strain the intrafusal muscle fibers (Richardson et al., 2006), increasing the availability of proprioceptive information, at least at the spinal cord level (Gandevia & Proske, 2012). This mechanism can likely explain the increased activation of the MG α‐motor units through the upregulation of motor unit recruitment and firing rate, as well as decreased recruitment and derecruitment thresholds. However, other factors like persistent inward currents could play an important role in motor unit threshold modulation (Heckman et al., 2008). Another strategy to enhance proprioceptive availability could involve visuomotor skills that activate the gamma loop (Ia afferents) for modulating sensory inputs (Perez et al., 2005). Additionally, increasing the net descending input in our setup may further support this goal. The visual information delivered could allow visuomotor integration (Unell et al., 2021) using the cerebellum's action as a comparator (Flament & Ebner, 1996), amplifying the proprioceptive information and net descending excitability.

Short‐term potentiation (Hennig, 2013) is a relevant peripheral condition that can also play a role in our study. Our setup induced repetitive muscle contractions that may increase presynaptic calcium concentration. This, in turn, leads to the opening of AMPA and NMDA receptors to increase the postsynaptic ionic conductance at the peripherical level (Hennig, 2013).

The coefficient of variance exhibited contrasting behavior between the limbs. This finding implies that our intervention stimulated motor unit synchronization (Semmler, 2002). However, force signal tracking showed worse synchronization compared to the non‐injured AT limb, leading to a decrease in their force coefficient of variance. It suggests impairment of motor unit synchronization in the repaired AT limb. This synchronization is useful for situations demanding rapid force development (Semmler, 2002).

Finally, we have not found evidence of tendon lengthening in this study. This is in accordance with the principle of no tendon lengthening during a critical mechanical phase, specifically during the early stage of AT healing. To set up our load conditions, we relied on our previous in vitro estimations to ensure no excessive stress at the suture‐tendon interface, which could potentially lead to silent tendon lengthening (De la Fuente, Cruz‐Montecinos, et al., 2017). Consequently, this case report presents novel evidence regarding an alternative intervention to enhance motor unit properties and activation. These properties are significantly affected during the first weeks of immobilization and can be responsible for chronic pathological muscle adaptation, which often leads to a low rate of sports return after experiencing an AT rupture.

As a research limitation, we can mention that the absence of measurements for H‐reflex and M‐response prevented the exploration of presynaptic and postsynaptic aspects during the patient's response to the intervention.

## 5 CONCLUSIONS

This single‐session neuromuscular biofeedback 12 days after AT surgery shows evidence of enhanced motor unit properties and activation without signs of AT lengthening when unresisted plantar flexion is performed in the repaired AT limb. Meanwhile, the non‐repaired AT limb requires lower demands of motor unit properties. These promising outcomes warrant further testing during the early phases of AT ruptures.

## AUTHOR CONTRIBUTIONS

The CRediT roles were; *Conceptualization*: Carlos De la Fuente, Rony Silvestre, Felipe P. Carpes; *Data curation*: Carlos De la Fuente, Felipe P. Carpes; *Formal analysis*: Carlos De la Fuente, Felipe P. Carpes; *Funding acquisition*: Carlos De la Fuente, Alejandro Neira; *Investigation*: Carlos De la Fuente, Rony Silvestre, Julio Botello, Alejandro Neira, Macarena Soldan, Felipe P. Carpes; *Methodology*: Carlos De la Fuente, Rony Silvestre, Felipe P. Carpes, Carlos De la Fuente, Felipe P. Carpes; *Resources*: Rony Silvestre; *Software*: Carlos De la Fuente, Rony Silvestre; *Supervision*: Carlos De la Fuente, Rony Silvestre, Felipe P. Carpes; *Validation*: Carlos De la Fuente, Rony Silvestre, Julio Botello, Alejandro Neira, Macarena Soldan, Felipe P. Carpes; *Visualization*: Carlos De la Fuente, Felipe P. Carpes; Roles/*Writing – original draft*: Carlos De la Fuente, Rony Silvestre, Julio Botello, Alejandro Neira, Macarena Soldan, Felipe P. Carpes; *Writing – review & editing*: Carlos De la Fuente, Rony Silvestre, Julio Botello, Alejandro Neira, Macarena Soldan, Felipe P. Carpes.

## FUNDING INFORMATION

Universidad Mayor (Santiago, Chile) funded this publication.

## CONFLICT OF INTEREST STATEMENT

The authors declare no conflicts of interest.

## ETHICS STATEMENT

The study was approved by the institutional review board from the MEDS Clinic (Santiago, Chile) and was conducted according to the Helsinki Declaration. The patient agreed to participate, provided written informed consent, and was anonymized for not tracking assumptions.

## Supporting information


**Data S1:** Supporting information

## Data Availability

Original datasets are available in the Researchgate repository of the first author https://www.researchgate.net/publication/374923237_Unique_Case_Study_Impact_of_Single‐Session_Neuromuscular_Biofeedback_on_Motor_Unit_Properties_Following_12‐days_of_Achilles_Tendon_Surgical_Repair_Matlab_data_after_sEMG_decomposition_and_raw_data.
