# Supplementary material for: Unique case study: Impact of single‐session neuromuscular biofeedback on motor unit properties following 12 days of Achilles tendon surgical repair
Source: Physiol Rep. 2024 Jan 9;12(1):e15868. doi: 10.14814/phy2.15868 (PMC10776338; doi:10.14814/phy2.15868)
Supplement: Supplementary file 1 — Data S1: Supporting information [file PHY2-12-e15868-s001.pdf]

6 | SUPPLEMENTARY MATERIAL

6.1 Basal assessment

Basal measurements were obtained prior to the intervention (Table 1). The AT resting angle was measured with the patient lying prone with the knee flexed at 90º (Carmont et al., 2015). The MG pennation angle and thickness were quantified with the patient resting in a prone position with 0º of knee extension and 20º of plantar flexion and using ultrasonography at the level where the sEMG sensor (Anvanti, Trigno, Delsys Inc., USA) was attached to the skin (Hermens et al., 2000). The superficial and deep aponeurosis were the anatomical references (Cho et al., 2014). A maximal voluntary isometric plantar flexion contraction (MVIC) was measured in the non-injured AT limb using a wireless load cell sensor (S-beam load cell, Delsys Inc., USA) with the patient seated with hip, knee, and ankle at 90º. The AT rupture score (ATRS) and the verbal response to a numerical rating pain scale of the patient were obtained 30 min prior to the intervention session start.

6.2 Data Acquisition and Processing

The AT length was measured using musculoskeletal ultrasonography with a 12 MHz linear transducer (Lumify S4-1, Philips, The Netherlands) and a 50 mm graded metric tape (Seca 201, Germany). The measurement was taken from the tip of the calcaneus to the myotendinous junction of the MG.

For sEMG decomposition assumptions, the patient was positioned prone with 30º knee flexion and 20º plantar flexion to unload the AT during the testing (De la Fuente et al., 2017). Considering a randomized order, a plantar flexion contraction was performed by each limb to elicit 30% of the maximal voluntary isometric contraction (MVIC) measured in the non-injured limb. This submaximal intensity was defined to protect the surgical repair from failure (de la Fuente et al., 2016). In addition, low-intensity muscle contractions reduce the superposition of motor unit action potentials, providing better decomposition outputs (LeFever and De Luca, 1982) and minimizing silent deformation at tendon-suture, a lengthening mechanism for the Dresden technique (de la Fuente et al., 2016). A uniaxial load cell (Delsys Inc., Boston, USA) was attached perpendicular at the metatarsal level of both limbs to register the force signals during the contractions to allow the control of a trapezoidal plantar flexion contraction at the target intensity, which was sustained for 20 s (isometric assessment) before and after the intervention session (Figure 1).

Two decomposition sEMG sensors (Galileo sensor, Delsys Inc., Boston, USA) recorded sEMG data from the repaired AT and non-injured limbs. We used 4-dry-electrodes for decomposition (four cylindroid probes of 0.5 mm diameter (De Luca et al., 2006)) and 4-dry-rectangular electrodes for

references, which were positioned after the skin cleaning and shaving. The electrodes were 99.9% of Ag. The inter-electrode distance was 2.5 mm. The sEMG signals for decomposition were acquired with an sEMG Trigno amplifier (Delsys Inc., Boston, USA). An analog-digital converter of 16-bit sampled the sEMG signals at 2222 Hz. Simple differential capture with dual stabilizing reference was used (De Luca et al., 2006). The common-mode rejection ratio was lower than -80 dB. The total noise was lower than 750 nV, and an analog band-pass filtered with a 20-250 Hz cut-off frequency. A reference electrode was located at the lateral side of the most prominent bulk of the MG (Figure 1). The quality of the signals (electric interferences and contact problems) was visually inspected before data acquisition.

After sEMG signal acquisition, the data were decomposed into motor unit action potential trains basis. The threshold accuracy was set at > 80% (Jeon et al., 2020). The decomposition algorithm was based on a template-matching approach enhanced by maximum a posteriori probability methods (LeFever and De Luca, 1982). The maximum a posteriori probability (Bayesian inference) allowed to estimate the occurrence of motor unit firing and waveforms (LeFever and De Luca, 1982). The firing rate time series were obtained by convoluting the impulse of firing trains with a 1-s Hanning window (Jeon et al., 2020). All estimations were made using the NeuroMap 1.0 software (Delsys Inc., Boston, USA).

6.3 Statistical analysis

Data distribution was checked using the Shapiro-Wilk and Levene tests ($\alpha = 5\%$). The motor unit properties, sEMG amplitude (mean root square from the 5-second plateau during the trapezoid contraction), force paradigm, and tendon length were described as the obtained values, and the absolute and percentage differences (Δ = without – with feedback intervention) prior to and after the intervention, were described for both in the AT repaired and non-injured limbs. The raster plot also was created. The space generated by the recruitment threshold and the peak firing rate was clustered (density-based spatial clustering of application with noise - DBSCAN) to understand the acquired motor unit strategies. The Epsilon neighborhood was 4. The epsilon parameter defines the searching radius around a core containing at least the minimum defined neighbors. The minimal number of neighbors in our study was 3. All descriptive statistics were conducted using Matlab software (Mathwork Inc., Natick, USA).

6.4 References

Carmont, M. R., Grävare Silbernagel, K., Brorsson, A., Olsson, N., Maffulli, N. and Karlsson, J. (2015). The Achilles tendon resting angle as an indirect measure of Achilles tendon length

following rupture, repair, and rehabilitation. *Asia Pac J Sports Med Arthrosc Rehabil Technol* 2, 49–55.

Cho, K. H., Lee, H. J. and Lee, W. H. (2014). Reliability of rehabilitative ultrasound imaging for the medial gastrocnemius muscle in poststroke patients. *Clin Physiol Funct Imaging* 34, 26–31.

De la Fuente, C., Carreño-Zillmann, G., Marambio, H. and Henríquez, H. (2016). Is the Dresden technique a mechanical design of choice suitable for the repair of middle third Achilles tendon ruptures? A biomechanical study. *Rev Esp Cir Ortop Traumatol* 60, 279–285.

De la Fuente, C. D., Cruz-Montecinos, C., Schimidt, H. L., Henríquez, H., Ruidiaz, S. and Carpes, F. P. (2017). Biomechanical properties of different techniques used in vitro for suturing mid-substance Achilles tendon ruptures. *Clin Biomech (Bristol, Avon)* 50, 78–83.

De Luca, C. J., Adam, A., Wotiz, R., Gilmore, L. D. and Nawab, S. H. (2006). Decomposition of surface EMG signals. *J Neurophysiol* 96, 1646–1657.

Hermens, H. J., Freriks, B., Disselhorst-Klug, C. and Rau, G. (2000). Development of recommendations for SEMG sensors and sensor placement procedures. *J Electromyogr Kinesiol* 10, 361–374.

Jeon, S., Miller, W. M. and Ye, X. (2020). A Comparison of Motor Unit Control Strategies between Two Different Isometric Tasks. *Int J Environ Res Public Health* 17, 2799.

LeFever, R. S. and De Luca, C. J. (1982). A procedure for decomposing the myoelectric signal into its constituent action potentials--Part I: Technique, theory, and implementation. *IEEE Trans Biomed Eng* 29, 149–157.
